# Identification and Functional Analysis of Flowering Related microRNAs in Common Wild Rice (*Oryza rufipogon* Griff.)

**DOI:** 10.1371/journal.pone.0082844

**Published:** 2013-12-30

**Authors:** Zongxiang Chen, FuLi Li, Songnan Yang, Yibo Dong, Qianhua Yuan, Feng Wang, Weimin Li, Ying Jiang, Shirong Jia, XinWu Pei

**Affiliations:** 1 Institute of Biotechnology, Chinese Academy of Agricultural Sciences, Beijing, China; 2 College of Agriculture Science, MOE Key Lab of Tropic Biological Resources, Hainan University, Haikou, China; 3 Rice Research Institute, Guangdong Academy of Agricultural Sciences, Guangzhou, China; 4 Department of Pathophysiology, Capital Medical University, Beijing, China; Cankiri Karatekin University, Turkey

## Abstract

**Background:**

MicroRNAs (miRNAs) is a class of non-coding RNAs involved in post- transcriptional control of gene expression, via degradation and/or translational inhibition. Six-hundred sixty-one rice miRNAs are known that are important in plant development. However, flowering-related miRNAs have not been characterized in *Oryza rufipogon* Griff. It was approved by supervision department of Guangdong wild rice protection. We analyzed flowering-related miRNAs in *O. rufipogon* using high-throughput sequencing (deep sequencing) to understand the changes that occurred during rice domestication, and to elucidate their functions in flowering.

**Results:**

Three *O. rufipogon* sRNA libraries, two vegetative stage (CWR-V1 and CWR-V2) and one flowering stage (CWR-F2) were sequenced using Illumina deep sequencing. A total of 20,156,098, 21,531,511 and 20,995,942 high quality sRNA reads were obtained from CWR-V1, CWR-V2 and CWR-F2, respectively, of which 3,448,185, 4,265,048 and 2,833,527 reads matched known miRNAs. We identified 512 known rice miRNAs in 214 miRNA families and predicted 290 new miRNAs. Targeted functional annotation, GO and KEGG pathway analyses predicted that 187 miRNAs regulate expression of flowering-related genes. Differential expression analysis of flowering-related miRNAs showed that: expression of 95 miRNAs varied significantly between the libraries, 66 are flowering-related miRNAs, such as oru-miR97, oru-miR117, oru-miR135, oru-miR137, et al. 17 are early-flowering -related miRNAs, including osa-miR160f, osa-miR164d, osa-miR167d, osa-miR169a, osa-miR172b, oru-miR4, et al., induced during the floral transition. Real-time PCR revealed the same expression patterns as deep sequencing. miRNAs targets were confirmed for cleavage by 5′-RACE in vivo, and were negatively regulated by miRNAs.

**Conclusions:**

This is the first investigation of flowering miRNAs in wild rice. The result indicates that variation in miRNAs occurred during rice domestication and lays a foundation for further study of phase change and flowering in *O. rufipogon*. Complicated regulatory networks mediated by multiple miRNAs regulate the expression of flowering genes that control the induction of flowering.

## Introduction

MicroRNAs (miRNAs), present widely in eukaryotes, are a class of endogenous, non-coding small RNAs (20–24 nt) that regulate gene expression by targeted protein-coding gene mRNA sequence-specific cleavage, translation repression or DNA methylation at the post-transcriptional level, often resulting in gene silencing [1]–[3]. miRNAs genes are transcribed by RNA polymerase II into long, specific, hairpin-structure primary transcripts (pri-miRNAs). miRNAs are generated from pri-miRNAs by Dicer-like1 (DCL1) cleavage with the help of the RNA-binding protein DAWDLE (DDL), the C_2_H_2_-zinc finger protein SERRATE (SE), the double -stranded RNA-binding protein HYPONASTIC LEAVES1 (HYL1) , and other factors. Mature miRNAs are methylated by the S-adenosyl methionine-dependent methyltransferase Hua Enhancer 1 (*HEN1*), and are incorporated into the *ARGONAUTE* (*AGO*) proteins to form the RNA-induced silencing complexes (RISC) that are involved in gene silencing [4], [5]. miRNAs have an important function in diverse biological and metabolic processes in plants, including hormonal regulation, defense responses, tissue development, phase transition, flowering, and adaptation to a variety of biotic and abiotic stresses [6]–[8].

Studies in *Arabidopsis* [9], rice [10], [11], *Ipomoea nil* [12], and the early-flowering mutant of trifoliate orange [13] have shown that miRNAs, such as miR156 and miR172, regulate the expression of developmental factors involved in flowering. MiR156, one of the most highly conserved plant microRNAs, is part of an intrinsic pathway for controlling the transition from vegetative growth to flowering in plants. The regulation of this pathway is based on changes in the miR156 content, miR156 declines from the vegetative stage to the reproductive stage, but the levels of its targets, *SPL* (Squamosa Promoter Binding Protein Like) transcriptional factors, increase during the same period. However, *SPLs* control the transition between the juvenile and flowering stages by regulating the expression of a class of MADS box genes, which induced flowering [14]–[16]. On the other hand, miR172 controls flowering time and floral organogenesis by regulating expression of the transcription factor gene *APETALA2* (*AP2*) and other *AP2-like* genes. Like *AP2* mutants, over-expressing of miR172 plants flower earlier and produce abnormal floral organs. The roles of miR172 and *AP2*-like genes are important in controlling flowering time in plants [17], miR172 is relatively well conserved in different plant species such *Arabidopsis*, maize and rice. A recent study has shown that miR172 acts downstream of miR156, and its expression is regulated by miR156. MiR156 and miR172 have inverse patterns of expression, miR156 declines while miR172 increases during the plant life cycle, and the miR156 target SPL9 promotes transcription of miR172b [15], [18]. MiR159 degrades its target MYB33 in the process of gibberellin-induced flowering in *Arabidopsis*, resulting in non-expression of LFY, delayed flowering in short days and male sterility [19]–[21]. However, over-expression of miR319, a sequence highly similar to miR159, decreases the levels of TCP transcription factors, then delays flowering in long days, stamen morphogenesis, and causes abortive stamens [22]. MiR169 represses C gene expression in sepals and petals that target the nuclear transcription factor NF-YA, may promote drought stress and induce *Arabidopsis* early flowering in long days [23], [24].

The silencing of Argonaute (*OsAGO1a,b,c,d*) [25] or Dicer-like (*OsDCL1a*; Os03g02970) [26] genes in rice mediated by small interfering RNAs (siRNAs) gave dwarf plants with short roots, floral variation and other gross developmental defects. These results show that *OsAGO1* and *OsDCL1* are key enzymes involved in miRNA synthesis in rice. Silencing these genes can lead to miRNA loss, which further emphasizes their important functions during growth and development in rice. Since the first rice miRNA was cloned [27], many rice miRNAs have been detected by deep sequencing, and 713 mature miRNAs have been identified and deposited in miRBase(v20) to date. Some of these miRNAs have been function analyzed in tissues such as inflorescence, stalks and seedings [28], [29], seeds [30], and spikelets [31]. Seventy-five pollen-specific miRNAs have been cloned [32], 19 miRNAs induced by heavy metals in Cd-induced rice seeding [33], and 32 new miRNAs, as well as 7 H_2_O_2_-induced miRNAs have been discovered by deep sequencing [34]. Rice plants over-expressing osa-miR396c or osa-miR393 show decreased salt and alkali stress tolerance [35], [36]. Over-expression of miR172 in rice can lead to spikelet loss, abnormal floral organs, and reduced fertility [11].

The perennial O. *rufipogon* (Common wild rice), which are considered to be the ancestor of Asian cultivated rice species, *Oryza sativa* L. can be utilized in cross breeding to improve rice cultivars [37], [38]. Very few investigations into genes involved in flowering have been conducted in common wild rice. Hagiwara et al. [39] studied nucleotide polymorphism in the *FT*-like genes *Hd3a* and *RFT1* in cultivated rice and *O. rufipogon*, and found that polymorphism of *Heading date 3a* (*Hd3a*) and *Rice Flowering Locus T1* (*RFT1*) at substituted, synonymous mutated sites and silent sites in wild rice was higher than the cultivated rice. Furthermore, polymorphism in *RFT1* at all sites was higher than *Hd3a*. Our previous analysis of genetic diversity in the photoperiod genes *Heading date 1* (*Hd1*), *Earlyheading date1* (*Ehd1*) and *OsGI* in common wild rice populations from different regions of China showed variation in *OsGI* introns between *O. rufipogon* and cultivated rice [40], [41].

Flowering is one of the most important agronomical traits. In a survey of *O. rufipogon* in China, the flowering time varied between different populations as well as within the same population were observed [42], [43]. Most common wild rice plants flower once a year, but some populations flower twice a year. Within individual populations of common wild rice in Guangxi and Guangdong, there are both single-flowering plants and double-flowering plants. Transplanting these plants to the experimental base in Lingshui, Hainan Province and a growth chamber in the laboratory indicated that the flowering trait was genetically stable [44].

In this study, common wild rice plants with different flowering times were collected from a single population growing in Gaozhou, Guangdong Province. Three sRNA libraries, the two vegetative stage libraries CWR-V1 and CWR-V2, and the flowering stage library CWR-F2, were sequenced using Illumina high-throughput sequencing technology. CWR-V1 was constructed from a group of single-flowering plants, and CWR-V2 and CWR-F2 were constructed from a group of plants that flowered twice a year. We found 512 known rice miRNAs and predicted another 290 new miRNAs specific to *O. rufipogon*. miRNA target functional annotation, revealed that 187 of the *O. rufipogon* miRNAs were flowering-related miRNAs. Differential expression analysis of flowering-related miRNAs in the three libraries showed that we have identified flowering-related miRNAs (F-miRNAs), as well as early-flowering-related miRNAs (E-miRNAs) that induced the variation in flowering between the two groups.

## Materials and Methods

### Plant materials

Common wild rice collected from Gaozhou, Guangdong Province,was approved by Guangdong province Agriculture Department,which is a supervision department of Guangdong wild rice and transplanted to Plant Growth Chamber at at day/night temperatures of 32°C/28°C (8 h day/16 h night) and relative humidity of 65% for our research.There are not field trials in this study, we only survey flowering time of wild rice in the original habitat.There were two groups of plants that differed in their flowering times in the same population, the single-flowering group flowered from October to December, and the double-flowering group flowered initially in April to June, and again from October to December. In late April, two libraries, CWR-V1 and CWR-V2, were constructed from leaves sampled in the vegetative stage of the single-flowering group and the double-flowering group, respectively. Library CWR-F2 was constructed from leaves in the flowering stage of the double-flowering group. The time interval between CWR-V2 and CWR-F2 was 36 days.

### Small RNA library construction and sequencing

Total RNA was extracted with TRIzol Reagent (Invitrogen, 15596-026) according to the manufacturer's instructions. RNA samples that met the requirements were used to construct the sRNA libraries. In brief, total RNA was fractionated by 15% polyacrylamide gel electrophoresis (PAGE), and small RNAs in the range of 18–30 nt were purified. After dephosphorylation and ligation of a pair of Solexa adaptors to the 5′ and 3′ ends, sRNAs were reverse-transcribed and amplified by PCR to produce the sequencing libraries. Solexa/Illumina deep sequencing was performed at the Beijing Genomics Institute (BGI).

### Bioinformatics analysis of deep sequencing data and differential miRNA expression analysis

The clean reads were mapped to the rice genome (MSU Rice Annotation Release 7.0) using the program SOAP (–s 8 -v 0 -r 2), and sequences perfectly matching the genome were used for further analyses. Sequences matching known rice rRNA, tRNA, snRNA, scRNA and snoRNA in the Rfam10.1 (http://www.sanger.ac.uk/software/Rfam) and NCBI GenBank database were discarded. sRNAs aligned to repetitive regions or assigned to mRNA exon and intron regions were also filtered out. Subsequently, the remaining sequences were analyzed by BLASTn searches against rice miRNA mature sequences and precursor structures that have been annotated in miRBase 18.0 (http://www.mirbase.org/), to identify known rice miRNAs and base mutation miRNAs in the libraries.

Genomic sequences 150 nt upstream and 150 nt downstream (approximately 320 bp) surrounding each unannotated sRNA were extracted for secondary structures analysis by MIREAP (https://sourceforge.net/projects/mireap/). The essential criteria were used for identifying new miRNA candidates in common wild rice [45].

### Prediction of potential miRNA targets and discovery of flowering miRNA families

Potential miRNA targets were predicted using the psRNA Target (http://plantgrn.noble.org/psRNATarget/) with default parameters as described previously [46]. The *Oryza sativa* TIGR genome cDNA OSA1 Release 5 (OSA1R5) was used as the genomic library for the target search.To investigate the biological processes regulated by miRNAs, we combined all of the miRNA targets in the three libraries and conducted Gene Ontology (GO) enrichment analysis and Kyoto Encyclopaedia of Genes and Genomes (KEGG) pathway annotations [47]. Candidate targets were analyzed by BLAST search against selected references in the rice genome using AgriGO Singular Enrichment Analysis (SEA) with the default parameters, and calculated the calibrated *P*-value. If the *P*-value was ≤0.05, the biological function of the targets could be confirmed. Furthermore, KEGG pathway was used to further confirm the metabolic and signal transduction pathways [48].

The differential expression of flowering-related miRNAs between pairs of libraries was demonstrated by plotting the relative fold-change against the identified miRNAs as follows:

1. miRNAs expression was normalized, normalized expression = actual miRNA/total of clean reads ×1,000,000. Following normalization, expression was set to 0.01 for miRNAs that were not expressed in one of the libraries.
2. The fold-change and *P*-values were calculated from the normalized expression. The log2-ratio figure was then generated [31].

Fold-change formula: fold-change (Log_2_CWR-V2/CWR-V1) = log_2_ (normalized expression of miRNA in CWR-V2/normalized expression in CWR-V1).

*P*-value formula:
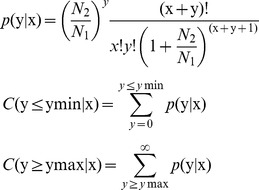
Where N_1_ is the total clean reads in CWR-V1, N_2_ is the total clean reads in CWR-V2, x is a miRNA reads surveyed in CWR-V1 and y is the miRNA reads in in CWR-V2.

If the miRNA fold-change was >1 or <−1, 0.01<*P*-value<0.05 represents a difference between the two libraries that was labeled “*”, and a *P*-value<0.01 represents a significant difference that was labeled “**”. The expression changes for CWR-F2/CWR-V1 and CWR-F2/CWR-V2 were analyzed by the same method.

### MiRNA cloning and stem-loop quantitative Real-Time PCR (qRT-PCR)

As described previously [49], miRNAs reverse transcription (RT) reactions were performed using specific stem-loop primers, the 3′ ends were complementary to both the 8 nt at the surveyed miRNA 3′ end and the 8 nt of the primer's 5′ end, which ensured the formation of a specific stem-loop structure. The last 10 nt of the forward PCR primer's 5′ end were consistent with the first 10 nt of the miRNA, and the reverse PCR primer was a universal primer that matched the stem-loop RT primers (Table S1). Approximately 2.5 µg of DNase I-treated total RNA was reverse-transcribed to cDNA using a TaqMan® MicroRNA Reverse Transcription Kit (Applied Biosystems) using a pulse reverse transcription program. cDNA was used as a template for miRNA cloning and quantitative real-time PCR (qRT-PCR).

Quantitative real-time PCR assays were performed to investigate the expression of new miRNAs in roots, stems, leaves and young panicles at the flowering stage. qRT-PCR was performed using SYBR® Select Master Mix (Applied Biosystems) on an ABI 7500 Real-Time System (Applied Biosystems). *OsActin* (GenBank Accession No. AB047313) was used as the endogenous control [50], and relative fold-changes in miRNA expression were calculated using the comparative threshold cycle (2^−ΔΔCT^) method as described [51]. All reactions were performed using one biological sample with three technical replicates.

### RNA ligase-mediated 5′-RACE

To investigate the cleavage sites in target mRNAs, we performed RNA ligase-mediated rapid amplification of 5′-cDNA ends (5′-RACE) using the FirstChoice® RLM-RACE kit (Ambion) according to the manufacturer's instructions. Briefly, polyA+ mRNA was enriched from 350 µg total RNA using an Oligolex® mRNA mini kit (Qiagen). mRNAs was directly ligated to the 5′-RACE RNA adapter (45 nt), and the ligation reaction was then reverse-transcribed into cDNA with an Oligo(dT)18 primer. Two 5′-RACE gene-specific outer and inner primers (Table S1) were used for each nested PCR. The PCR products were separated by AGE and cloned into the pEASY-T3 vector for sequencing.

## Results and Discussion

### The distribution of sRNA categories in the deep sequencing datasets

**Figure 1:**
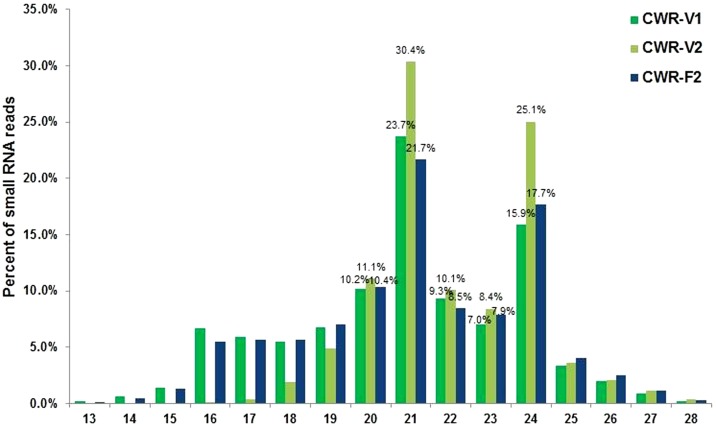
Length distribution of sRNAs in the three libraries at different developmental stages.

Sequencing of the CWR-V1, CWR-V2, and CWR-F2 libraries generated 23,850,262, 21,860,151 and 24,317,962 raw reads, respectively, of which 20,156,098, 21,531,511 and 20,995,942 corresponding genome-matched clean reads from the same three libraries, respectively (Table 1). The length distributions of these high quality small RNAs from the individual libraries showed that more than 60% of the sRNAs were between 20 and 24 nt (Figure 1), and the fraction of small RNAs in CWR-V2 was 85.14%, which is consistent with the representative size range of Dicer-like (DCL) cleavage products [52]. sRNA sequences with lengths of 21 nt were found significantly more frequently than those with other lengths, accounting for 23.73%, 30.41% and 21.70% in the three datasets, respectively. However, different lengths of miRNA are produced by cleavage mediated by different kinds of DCLs. Almost all conserved miRNAs were the canonical 21 nt DCL1 product, and DCL1 may cleave precisely under the action of DCL2 or DCL3. The precursors are cleaved by DCL1 and DCL3 to generate a class of 23–25 nt miRNAs, called long miRNAs. Certain miRNA precursors might generate 22 nt miRNAs, presumably as a result of the action of DCL2 [3].

**Table 1:** Distribution of different sRNA categories in the different libraries.

Category	CWR-V1		CWR-V2		CWR-F2	
	Unique(%)	Reads(%)	Unique(%)	Reads(%)	Unique(%)	Reads(%)
raw reads	—	23850262	—	21860151	—	24317962
high quality	—	23767886	—	21792329	24230246	
clean reads^a^	3081097(100)	20156098(100)	3493671(100)	21531511(100)	3520447(100)	20995942(100)
exon_antisense	37918(1.2)	112669(0.6)	41404(1.2)	216290(1.00)	66389(1.9)	207313(1.0)
exon_sense	259552(8.4)	533039(2.6)	143591(4.1)	389764(1.8)	258146(7.3)	537402(2.6)
intron_antisense	38081(1.2)	108493(0.5)	44737(1.3)	199531(0.9)	55011(1.6)	147424(0.7)
intron_sense	60484(2.0)	151224(0.8)	57942(1.7)	267035(1.2)	72396(2.1)	184186(0.9)
miRNA^b^	6536(0.2)	3448185(17.1)	5757(0.2)	4265048(19.8)	6429(0.2)	2833527(13.5)
rRNA	251603(8.2)	9501946(47.1)	258938(7.4)	7094006(32.9)	287817(8.2)	10111526(48.2)
repeat	865259(28.1)	1964625(9.7)	868423(24.9)	2624071(12.2)	822129(23.4)	1681420(8.0)
snRNA	3941(0.1)	18076(0.1)	3654(0.1)	16987(0.1)	4960(0.1)	19862(0.1)
snoRNA	3586(0.1)	7471(0.0)	3738(0.1)	11091(0.1)	4640(0.1)	10080(0.0)
tRNA	35389(1.2)	1480266(7.3)	31469(0.9)	1928031(9.0)	39198(1.1)	1705003(8.1)
unann^c^	1518748(49.3)	2830104(14.0)	2034018(58.2)	4519657(21.0)	1903332(54.1)	3558199(16.9)

^a^ The number of genome-matched clean reads of high quality sRNAs.

^b^ The number of reads included the known rice miRNAs ±2 variants.

^c^ Contains all of the non-annotated sequences that possibly include new miRNAs and base mutations in known miRNAs.

The genome-matched sRNA covered various categories of sRNA, including miRNA, siRNA, rRNA, tRNA, snRNA, snoRNA, and repeats and degradation fragments derived from exons and introns (Table 1). Besides unannotated RNAs, rRNA was the most abundant class of identified sRNAs, accounting for 47.1%, 32.9% and 48.2% from the CWR-V1, CWR-V2, and CWR-F2 libraries, respectively. Similar to previous reports in rice [30], [32], the numbers of known rice miRNA reads were 3,448,185, 4,265,048, and 2,833,527, respectively, accounting for 17.1%, 19.8% and 13.5% of all clean reads in the three libraries, but the percentages of unique miRNAs were only 0.2%, suggesting that mature miRNAs were particularly enriched in our libraries and might be the most abundant class of sRNAs regulated factors at the post-transcriptional level. The number of miRNAs expressed in the flowering stage library CWR-F2 was lower than in the two vegetative stage libraries CWR-V1 and CWR-V2, and the number of unique miRNAs was not significantly different between the libraries. We hypothesize that highly expressed miRNAs benefit normal development in *O. rufipogon* by prolonging the vegetative stage, and some non-conserved miRNAs with lower expression levels may displace the original conserved miRNAs at a specific stage of flowering, resulting in a reduction in the total number of miRNA reads while the unique fraction remains unchanged. Furthermore, many sRNAs were unannotated because of spatio-temporal-specific expression, genome differences between wild rice and cultivated rice, sequencing errors, etc. These sRNAs might contain new miRNAs specific to *O. rufipogon*, known miRNAs with base mutations, and/or other kinds of small RNA molecules.

### Known rice miRNAs expressed in *O. rufipogon*

A sequence similarity search in miRBase 18.0 found 512 known rice miRNAs corresponding to 214 miRNA families in the libraries. The miRNAs were generated from 448 miRNA precursors, and 80 families had more than one miRNA member and 134 families only had one (Table S2). Conservative analysis of known miRNAs showed that 153 miRNAs corresponding to 27 miRNA families were from Group I that are well conserved among diverse angiosperms, and all 22 miRNA families were conserved in both *Arabidopsis* and rice [53]. Thirty miRNAs corresponding to 18 miRNA families were in the monocot-specific Group II, and 328 miRNAs corresponding to 168 miRNA families were in Group III, which is specific to rice. However, only one mature miRNA, miR419, from Group IV, was detected by computational prediction in the CWR-F2 library. The remaining 149 known rice miRNAs were undiscovered in miRBase, and possible reasons for this are as follows: (1) sRNAs from wild rice do not match sequences in the rice genome due to genomic differences between *O. rufipogon* and *O. sativa*; (2) some miRNAs were identified by bioinformatic prediction, not deep sequencing; (3) the expression of miRNAs with spatio-temporal specificities or that are induced by stress was too low to be represented in the sequenced libraries; and (4) the miRNAs really do not exist in the wild rice genome, and represent rice-specific miRNAs that arose during evolution.

From the distribution of miRNAs in the three libraries, there were 442, 423 and 473 mature miRNAs out of the total of 512 expressed in CWR-V1, CWR-V2, and CWR-F2, respectively. The expression of miRNAs in the two vegetative libraries, CWR-V1, CWR-V2, differed from that in the flowering stage library, CWR-F2; 39 miRNAs were expressed only during the vegetative stage, 42 miRNAs were expressed only during the flowering stage, and 431 miRNAs were expressed in both stages, indicating that most of the miRNAs are expressed throughout the entire plant lifecycle, but that only a few are specific to a certain development stage. However, for flowering time, 22 miRNAs were expressed only in the single-flowering group, 70 miRNAs were expressed only in the double-flowering group, and 420 miRNAs were expressed in both groups. There were also 10 vegetative-specific miRNAs, 42 flowering-specific miRNAs, and 18 non-specific miRNAs in the 70 vegetative miRNAs in the double-flowering group.

Similar to previous deep sequencing reports in rice [28], [30], [34], the expression of conserved miRNAs was considerably higher than that of non-conserved miRNAs. The miR156 family tops the list of conserved miRNAs, followed by the miR168, miR66, miR167 and miR528 families, the total expression of these families accounted for over 90% of the known miRNAs in the three libraries. The most highly expressed miRNA, the miR156 family, accounted for 42.4%, 64.1% and 66.9% of miRNAs in CWR-V1, CWR-V2, and CWR-F2, respectively. In addition, the expression of diverse members of the same family was very different. For example, the expression of miR156a-miR156j was in the millions of reads, while there were only 15 and 1953 reads specific for miR156k and miR156l, respectively. The number of miR172a and miR172d-specific reads were 4805 and 11717, but the maximum expression of miR172b was 74 reads, and there were only three miR172c-specific reads. The expression of the other non-conserved miRNA families is irregular, with the expression of highly expressed miRNAs, such as miR444, miR812, in the thousands of reads. Weakly expressed miRNAs had <10 reads, examples are miR1319, miR1431, and miR1441. The Group II miR528 family displayed a large difference between the three libraries, the number of expressed reads were 895765, 2122, and 54883 in CWR-V1, CWR-V2, and CWR-F2, respectively. Conserved miRNAs generally had different levels of expression in the two species, the most highly expressed member of a given family might play a leading functional role in regulating one or more genes or gene families.

The high sensitivity of the deep sequencing technology makes it feasible to investigate the distributions and numbers of the unique sequences along the miRNA precursors. We identified only 48 complementary miRNAs* (3p sequences) corresponding to the miRNAs from 512 mature miRNA sequences, and miR319a had two different miRNAs*. Most miRNAs* were expressed in less than 20 reads, because the miRNAs* are degraded during the process of miRNA synthesis. But we also found that the expression of 21 miRNAs* generated from pre-miRNAs was higher than their complementary miRNAs (Figure S1A). An example is miR1425 in CWR-F2, its complementary miR1425-3p had 4312 reads, but the mature miRNA had only 1062 reads. The same phenomena was also observed for miR1846d, miR1853, miR1860, and others. This observed degradation of miRNAs* is a gradual process, and the miRNAs* might be regarded as authentic miRNA sequences because both the miRNA and miRNA* can function simultaneously in regulating gene expression. Similar to the study of H_2_O_2_-induced miRNAs in rice, many variants were observed within a ±2 nt length difference from the mature miRNA sequences, and some of them were more abundant than the corresponding mature miRNAs [34]. For example, the highest expression of all the variants of miR1425 is one that has a uridine (U) at the 3′ end of the mature miRNAs sequence, not the mature sequence, as well as miR1846d*. Perhaps this simply indicates that annotated miRNAs sequences are not necessarily real rice miRNAs, and that there is a complex mechanism of miRNAs biogenesis that includes multiple enzymes. Furthermore, 14 single miRNA precursors could generate more than one mature miRNAs (Figure S1B). For example, pre-miR159a generated two different mature miRNAs sequences, miR159a.1, which is expressed at a higher level than miR159a.2. Pre-miR1850 and pre-miR444d both formed three mature miRNA sequences, and pre-miR319a formed two miRNAs* sequences corresponding to miR319a-5p, miR319a-3p and miR319a-3p.2. The expression of mature miRNA sequences from the same precursors but pre-miR444d has a big difference, most expressed less than 100, and couldn't find their complementary miRNAs* sequences. The weakly expressed miRNA could be a by-product of DCL cleavage because of variation in the cleavage sites in miRNA precursors, and the highly expressed miRNAs are the authentic miRNAs sequences.

### Newly-identified miRNAs in *O. rufipogon*

**Figure 2:**
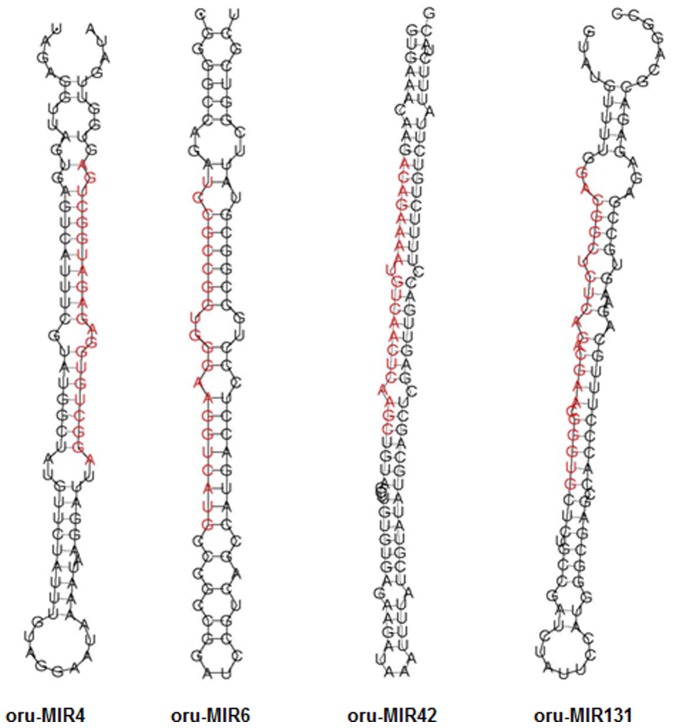
Examples of stem-loop hairpin secondary structures of predicted new miRNA precursors. Segments corresponding to the mature miRNAs are shown in red.

The genomic sequences surrounding each unannotated sRNA were extracted for secondary structure analysis by MIREAP, we predicted 290 new miRNA candidates generated from 337 miRNA precursor loci in three libraries (Table S3). Secondary analysis showed that the new miRNA precursors formed specific stem-loop structures (Figure 2), and the lengths varied from 68 to 374 bp, with an average of 184 bp. The average MEF value was −68.7 kcal/mol, with a range of −18.52 to −251.0 kcal/mol. The new miRNA precursors were located in vastly different positions, and the number of precursors on the sense strand of the genome was 151, with 139 located on the antisense strand. Being consistent with previous studies [54], 169 new miRNA loci were located in intergenic regions, 37, 34 and four were located in exons, the intron-exon junctions, and the UTRs (both 5′ and 3′), respectively. Exonic miRNAs were discovered in the study of rice H_2_O_2_-induced miRNAs [34]. Also, 46 new miRNAs have been located in the introns of protein-coding genes, these intronic miRNAs could be mirtrons that do not rely on *DCL* cleavage, but rather are spliced and debranched by lariat debranching enzyme (Ldbr), after which they fold into pre-miRNA hairpins and are processed to form mature miRNAs, a known example in rice is miR1429.2 [55].

Most new miRNAs originate from single genetic loci, with only a single precursors present in the genome. However, 20 new miRNAs were found to be originated from more than one genetic locus, 16 of these new miRNAs were uniquely expressed in our libraries. For example, oru-miR1, oru-miR37 and oru-miR53 both had three loci in CWR-V1, oru-miR105 and oru-miR188 had two loci in CWR-V2 and CWR-F2, and oru-miR64 had three loci in all three libraries. More interestingly, oru-miR199 only had one locus in CWR-F2, but had two loci in CWR-V2. Base analysis revealed that the 5′ end base was mainly U (34.5%), and this was probably because AGO1 has the strongest binding affinity for sRNAs that initiate with U [2]. We also found 20 corresponding miRNA* sequences out of 290 new expressed miRNAs, miRNAs and their complementary miRNA* sequences were located on two different arms of the precursor sequences (Figure S2). In this case, the highly expressed sequences were regarded as mature miRNA sequences, and the complementary weakly expressed sequences were the corresponding miRNA* sequences resulting from miRNA* degradation after the process of DCL1 cleavage. As can also be seen from Figure S2, there were 16 mature sequences out of 20 miRNAs with complementary miRNAs* generated from the 5′ end of the precursors, while the other mature miRNA sequences, oru-miR47, oru-miR75, oru-miR216 and oru-miR287, were located at the 3′ end of the precursors.

**Figure 3:**
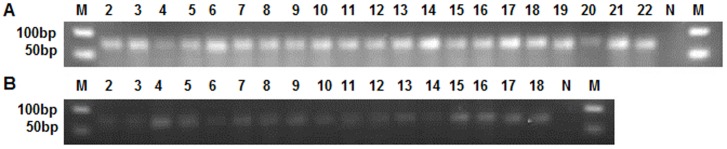
TA-cloning analysis of 33 new miRNAs that have complementary miRNAs* or that had >100 reads. (A) Lanes 2–6 are the positive controls, known rice miRNAs osa-miR156d, osa-miR166c, osa-miR167d, osa-miR168a, and osa-miR172a. Lanes 7–22; osu-miR1, osu-miR10, osu-miR105, osu-miR123, osu-miR131, osu-miR147, osu-miR150, osu-miR174, osu-miR18, osu-miR180, osu-miR216, osu-miR223, osu-miR238, osu-miR253, osu-miR260, and osu-miR266. (B) Lanes 2–18 are osu-miR284, osu-miR287, osu-miR35, osu-miR4, osu-miR47, osu-miR51, osu-miR6, osu-miR61, osu-miR62, osu-miR64, osu-miR68, osu-miR73, osu-miR75, osu-miR86, osu-miR88, osu-miR96, and osu-miR99. N; negative control; M; 50 bp DNA Ladder size marker.

**Figure 4:**
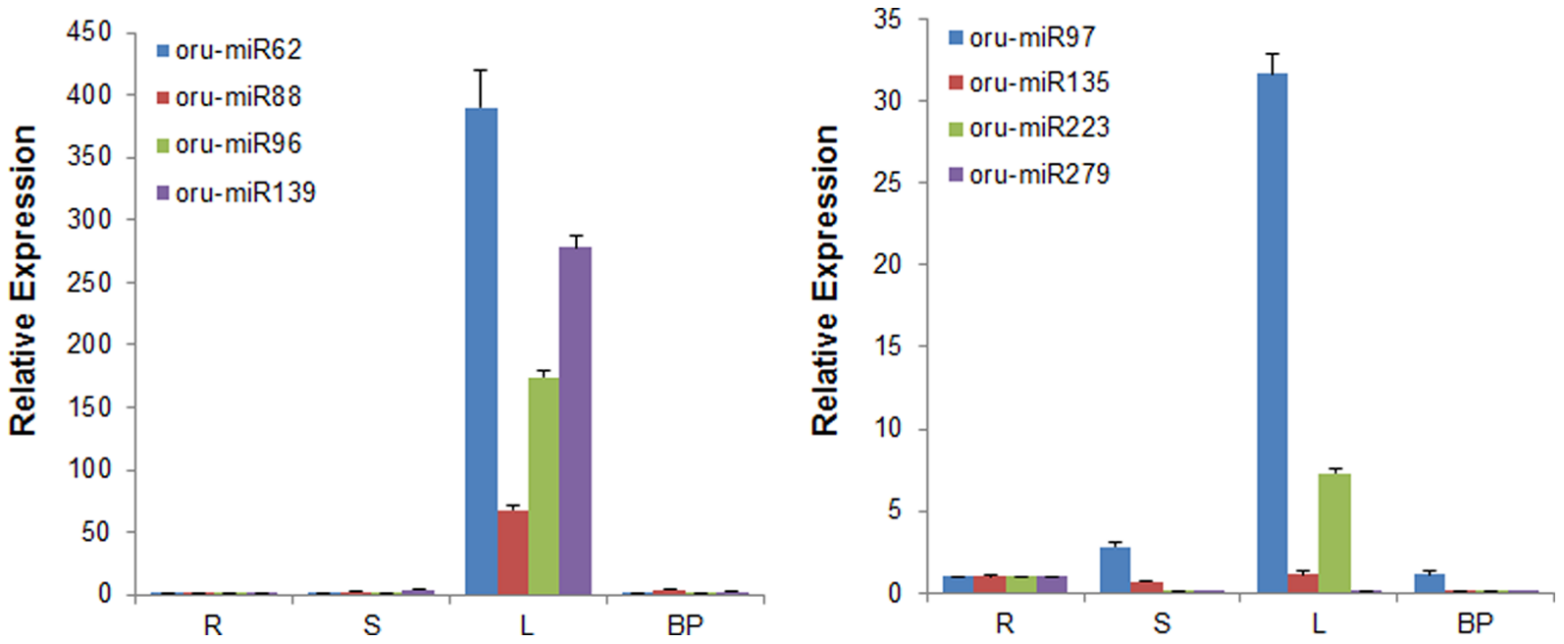
Quantitative real-time RT-PCR analysis of the relative expression of new miRNAs in different rice tissues. OSActin was used as a housekeeping gene control. Real-time PCR experiments were conducted using the primers given in Table S1. The data represent the mean values ±SD of three replicates. R: root, S: stem, L: leaf, BP: booting panicle.

From the distribution of 290 new oru-miRNAs in the three libraries, there were 102, 127 and 137 oru-miRNAs in CWR-V1, CWR-V2, and CWR-F2, respectively. Of these, 233 oru-miRNAs were expressed in a single library, 19 oru-miRNAs were expressed in all three libraries, and the remaining 38 were expressed in two of the libraries. With respect to developmental stages, 148 oru-miRNAs were expressed during the vegetative stage, 85 oru-miRNAs were expressed during the flowering stage, and the other 57 oru-miRNAs were expressed in both stages. For flowering times, 68 oru-miRNAs were expressed in the single-flowering group, 188 oru-miRNAs were expressed in the double-flowering group, and 34 oru-miRNAs were expressed in both groups. The distributional difference among oru-miRNAs is consistent with known rice miRNAs as above. Comparing the expression of the new oru-miRNAs with known rice miRNAs showed that oru-miRNAs were expressed at a much lower level than were known rice miRNAs, most had <100 reads, and the highest level of expression was for oru-miR51, which had only 1154 reads in CWR-F2.Selected new miRNAs with complementary miRNAs*, or those that had >100 reads, were amplified for cloning and sequencing. Five known rice miRNAs (osa-miR156d, osa-miR166c, osa-miR167d, osa-miR168a, and osa-miR172a) as the positive controls, and 33 tested oru-miRNAs all gave a single PCR band with a length of approximately 70 bp (if the length of miRNA is 21 nt, the amplified product will be 69 bp) on the gel (Figure 3).The miRNA sequences were consistent with the deep sequencing results. This suggests that the predicted new oru-miRNAs are authentic miRNAs specific to common wild rice.Expression analysis of new miRNAs in roots, stems, leaves, and booting panicles in the flowering stage indicated that most oru-miRNAs are mainly expressed in leaves (Figure 4). Oru-miR279 was mainly expressed in roots, expressed at lower levels in leaves, and barely expressed in stems and booting panicles. These results indicated that new oru-miRNAs had tissue-specific patterns of expression in *O. rufipogon*, and are not present in rice.

### Target prediction and functional analysis of known and new miRNAs

Four-hundred fifty-four of 512 known rice miRNAs and 226 of 290 new oru-miRNAs had a total of 8,791 potential targets, with an average of 12.9 targets/miRNA was predicted. Most miRNA families have multiple target sites, indicating that these miRNAs play more than one role in plant development. On the other hand, a single gene may be also targeted by several miRNAs, these miRNAs map to the same cDNA at different sites, and cleave the mRNA into different-sized fragments.

**Figure 5:**
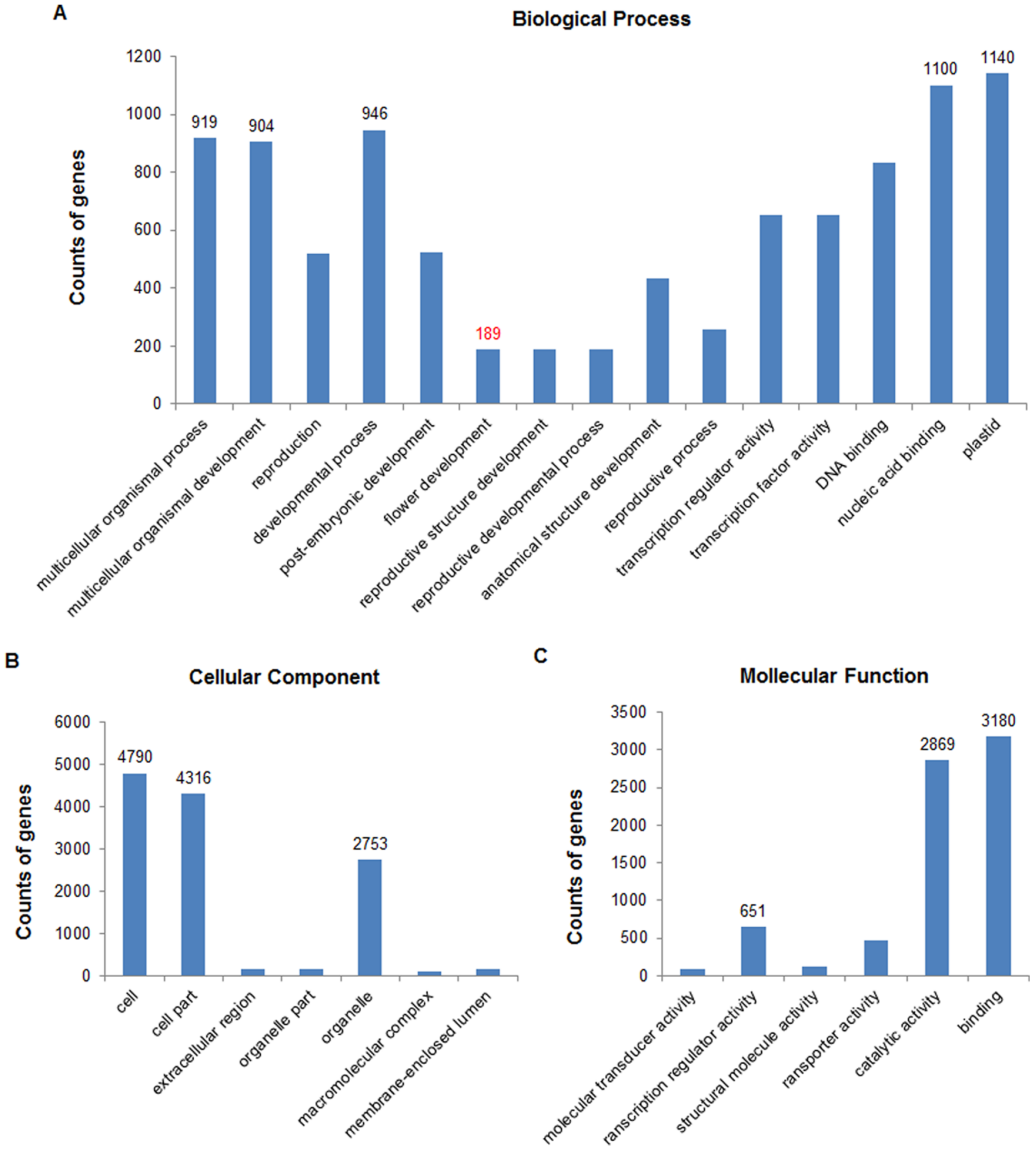
Gene ontology (GO) categories of target genes of known and new miRNA families. Categorization of miRNA target genes was performed according to the three GO domains; Biological Process (A), Cellular Component (B), and Molecular Function (C).

miRNAs targets were annotated using GO enrichment analysis [47], [48]. The 8,791 predicted targets were analyzed by BLASTn searches against selected references in *Oryza sativa* MSU 7.0 (non-TE), 7,388 of the targets were mapped to the 34,296 genomic genes, and the number of significant GO terms was 15 (Figure 5). For Biological Process (Figure 5A), these genes were classified into 15 categories, One-hundred and eighty-nine miRNA targets (189) matched with flower development (GO:0009908). These results suggest that miRNAs are involved in a wide range of physiological functions, and some miRNA targets associated with flowering. Cellular Component categories indicated that the miRNA targets were related to seven cellular parts (Figure 5B), and the three most frequent terms were cell (4790), cell part (4316) and organelle (2753). The three most highly represented GO terms out of six categories in Molecular Function were binding (3180), catalytic activity (2869) and transcription regulator activity (651) (Figure 5C). In addition, these targets were further annotated using KEGG pathway analysis. A total of 254 pathways were found, some of which were consistent with biological processes revealed by GO. The five most frequent pathways were metabolic pathways, biosynthesis of secondary metabolites, plant-pathogen interaction, RNA degradation, and ribosome biogenesis in eukaryotes. Importantly, we found that 32 miRNA targets were involved in the rice circadian rhythm in our KEGG pathway analysis, the pathways that contained rhythmic regulated genes included UV-B protection, cell elongation, photoperiodic flowering, and antenna proteins. Only seven out of 32 targets regulated the process of UV-B protection, the other 25 targets acted upstream of photoperiodic flowering (Figure S3).

### Identification of flowering related miRNAs and target validation

Functional annotation and sequence homology analysis indicated that 54 miRNAs targets were directly associated with flowering in rice. For example, *Os07g30250*, the target of both miR1436 and miR818, was predicted to encode a putative *RFT1* protein, which was reported to be a rice florigen that promoted flowering in rice [56]. The target of miR2864.1, *Os09g03610*, was predicted to be a homolog of the *FCA* protein that controlled flowering time [57]. The target of miR811, *Os02g26210.1*, was predicted to be a flowering promoting factor-like 1, and the target of miR819 was predicted to induce early flowering. In addition, GO functional analysis revealed that out of 189 miRNA targets related to flower development, five (*Os09g03610.1-Os09g03610.4* and *Os02g26210.1*) were also found by functional annotation, and KEGG pathway analysis indicated that 25 targets are involved in the rice circadian rhythm to regulate photoperiodic flowering. Both of these analyses identified *Os11g34460.2* that is the target of the miR1884 and miR2123 families, and is predicted to be an *OsFBO10* gene involved in the process of photoperiodic flowering and the control of flowering time and circadian rhythms in plants [58]. We therefore concluded that 262 predicted miRNA targets were closely related to flower development in *O. rufipogon* (Table S4). Based on the interaction between miRNAs and their targets, 146 known rice miRNAs and 41 new oru-miRNAs are involved in flower development in *O. rufipogon*.

In our study, the wild rice plants collected from Gaozhou, Guangdong Province, could be divided into two different groups, one of which flowers once a year, and the other that flowers twice a year. The variants that flower twice yearly must result from an endogenous genetic change, or a change in metabolic regulation. As one of the most important post-transcriptional regulators of gene expression, miRNAs actively participate in the flowering time variation, and could induce early flowering. Also, the formation and development of floral organs during flowering are related to regulatory function of miRNAs. Thus, we know that 187 flowering related miRNAs included not only early-flowering miRNAs that play a role in the flowering variation observed between the CWR-V1 plants, and the CWR-V2 and CWR-F2 plants, but also included flowering-related miRNAs from the flowering stage that differed between CWR-V2 and CWR-F2.

**Figure 6:**
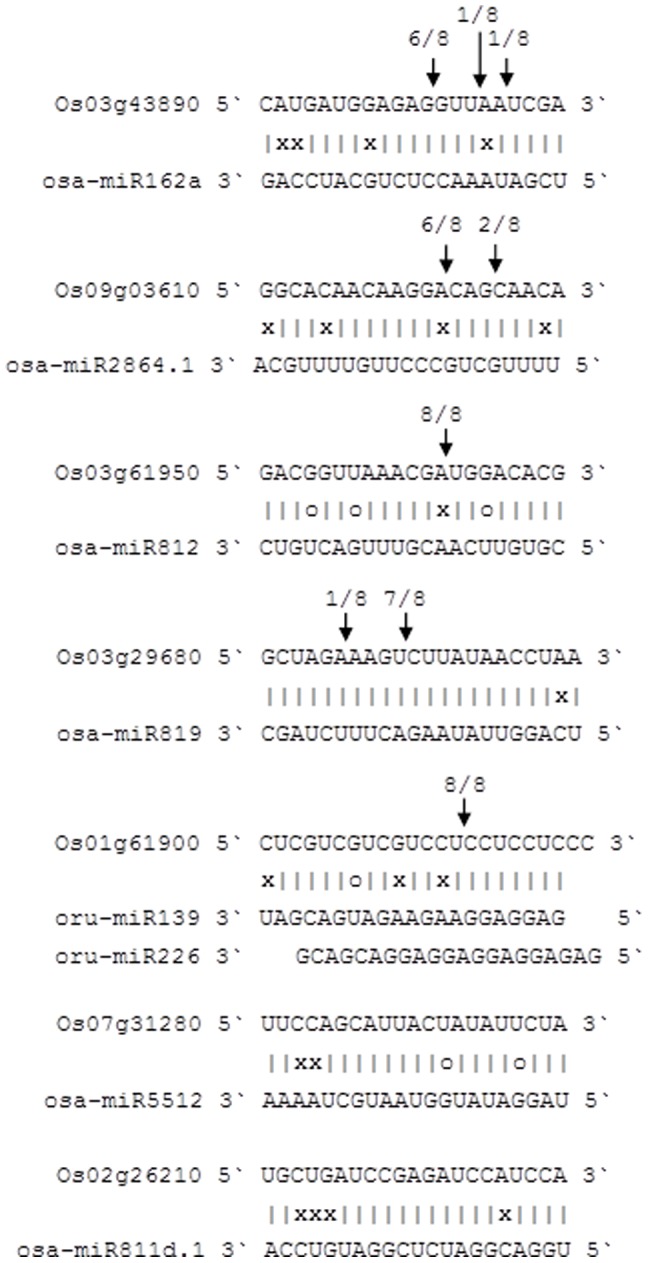
Mapping target mRNA cleavage sites by RNA ligase-mediated 5′-RACE. The arrows indicate the cleavage sites and the numbers show the frequency of clones sequenced.

Sequencing nested PCR products amplified by 5′-RACE outer and inner primers revealed that cleavage sites were located in the complementary region between miRNAs and their targets, and that some miRNAs had multiple cleavage sites with different relative activities (Figure 6). The target of osa-miR162a, *Os03g43890*, is predicted to be a *WD*-type transcription factor in the anthocyanin biosynthesis pathway, there are three cleavage sites with different relative activities in the complementary region, and the site with the highest activity is located in positions 9–10 at the 5′ end of the miRNA. The *FCA* gene, *Os09g03610*, that controls flowering time is cleaved by osa-miR2864.1 at two different sites. The osa-miR812 family cleaves the *ZTL* factor gene, *Os03g61950*, which plays a role in regulating circadian rhythms, and the osa-miR819 family also had two different activity sites to cleave *Os03g29680*, a gene encoding a putative early-flowering protein. In addition, as above, a single gene may be also targeted by several miRNAs. For example, *Os01g61900*, a homolog of *TIMING OF CAB 1* (*TOC1*) that encodes a CCT-motif family protein, is cleaved by oru-miR139 and oru-miR226 at the same position. As a nuclear-localized putative transcription factor (Figure S3), *TOC1* regulates flowering in circadian rhythms [59]. On the other hand, not all miRNAs actually cleave their predicted targets, for example, the osa-miR5512 and osa-miR819 families do not cleave their predicted targets *Os07g31280* and *Os03g29680*, respectively. The reason for this may be as follows: (1) miRNAs experienced base mutation during evolution, resulting in a change in their targets; (2) miRNAs can be expressed at very low levels or are tissue-specific, so the cleaved fragments of the target mRNA are below the level of detection; (3) the mechanisms of action are translation repression or DNA methylation, but not mRNA sequence-specific cleavage; (4) experimental errors, such as low quality RNA or inappropriate 5′-RACE primers. This further illustrates that a series of experiments needs to be conducted to verify the identification of miRNA targets in addition to bioinformatic prediction.

### Expression patterns of flowering-related miRNAs during phase change

We carried out significant difference expression analyses of flowering-related miRNAs among the three sRNA libraries and calculated the fold-changes and *P*-values between pairs of libraries (Table S5). We concluded that 66 flowering miRNAs out of 187 were expressed very weakly (≤20 reads) in all three libraries. We were unable to calculate the P-values and abandoned difference analysis for these miRNAs. In the rest of 121 miRNAs, 26 flowering-related miRNAs in the three libraries showed no significant differences, the remaining 95 flowering-related miRNAs showed significant differences between the three libraries. There were only 10 new oru-miRNAs present among the 95 flowering-related miRNAs. Oru-miR137 and oru-miR97 were expressed in two libraries, while oru-miR117, oru-miR135, oru-miR139, oru-miR177, oru-miR180, oru-miR222, oru-miR223, and oru-miR4 were expressed in a single library. Two known rice miRNAs out of 85, osa-miR3979 (CWR-V2) and osa-miR5338 (CWR-F2), were expressed in one library, but the remaining miRNAs had varying levels of expression in all three libraries. Sixty-two flowering miRNAs out of 95 showed significant differences in expression between the CWR-V1 and CWR-V2, 42 miRNAs had increased expression, and 20 had reduced levels of expression (Table S6). Forty-four out of 95 flowering miRNAs showed significant differences between CWR-V1 and CWR-F2, and of these, 41 were highly significant, 17 miRNAs had increased expression, and 27 had reduced levels of expression (Table S6). There were 66 flowering related miRNAs out of 95 showed significant differences between CWR-V2 and CWR-F2, 20 miRNAs showed increased expression, and 46 had reduced expression (Table S6). Two differential expression analyses comparing the two developmental stages both showed that most flowering-related miRNA presented a declining trend during the transition from the vegetative stage to the flowering stage, and the trend was even more obvious in the double-flowering group. The total number of reads that matched to known rice miRNAs was the lowest in the flowering stage, further suggesting that miRNAs are highly expressed in the vegetative stage, but are expressed at a lower level during the flowering stage.

The expression of 66 flowering related miRNAs that showed significant differences between CWR-V2 and CWR-F2 and that changed markedly during the development of floral organs from the vegetative stage to the flowering stage were regarded as flowering-related miRNAs, these were designated as ‘F-miRNAs’. The 17 early-flowering miRNAs that showed significant differences in expression levels between CWR-V1 and CWR-F2, but not between CWR-V2 and CWR-F2, were designated as ‘E-miRNAs’. The E-miRNAs induced the double-flowering group to flower earlier, resulting in flowering variation. The functions of the last 12 flowering miRNAs with significant differences during developmental stages between CWR-V1 and CWR-V2 were not clear in *O. rufipogon*, this group were designated as ‘N-miRNAs’. The reasons for the differences were as follows: (1) all N-miRNAs were in Group I (known rice miRNA families) except for the highly conserved osa-miR819h-3; (2) the miRNAs had significantly different expression levels in different periods of the long vegetative stage in *O. rufipogon*. Interestingly, most of single-library-expressed flowering miRNAs are F-miRNAs, except for oru-miR4, which is an E-miRNA. This further indicates that both F-miRNAs and E-miRNAs display spatio-temporal-specific expression patterns, but that N-miRNAs are highly conserved and have functional roles similar to housekeeping genes.

**Figure 7:**
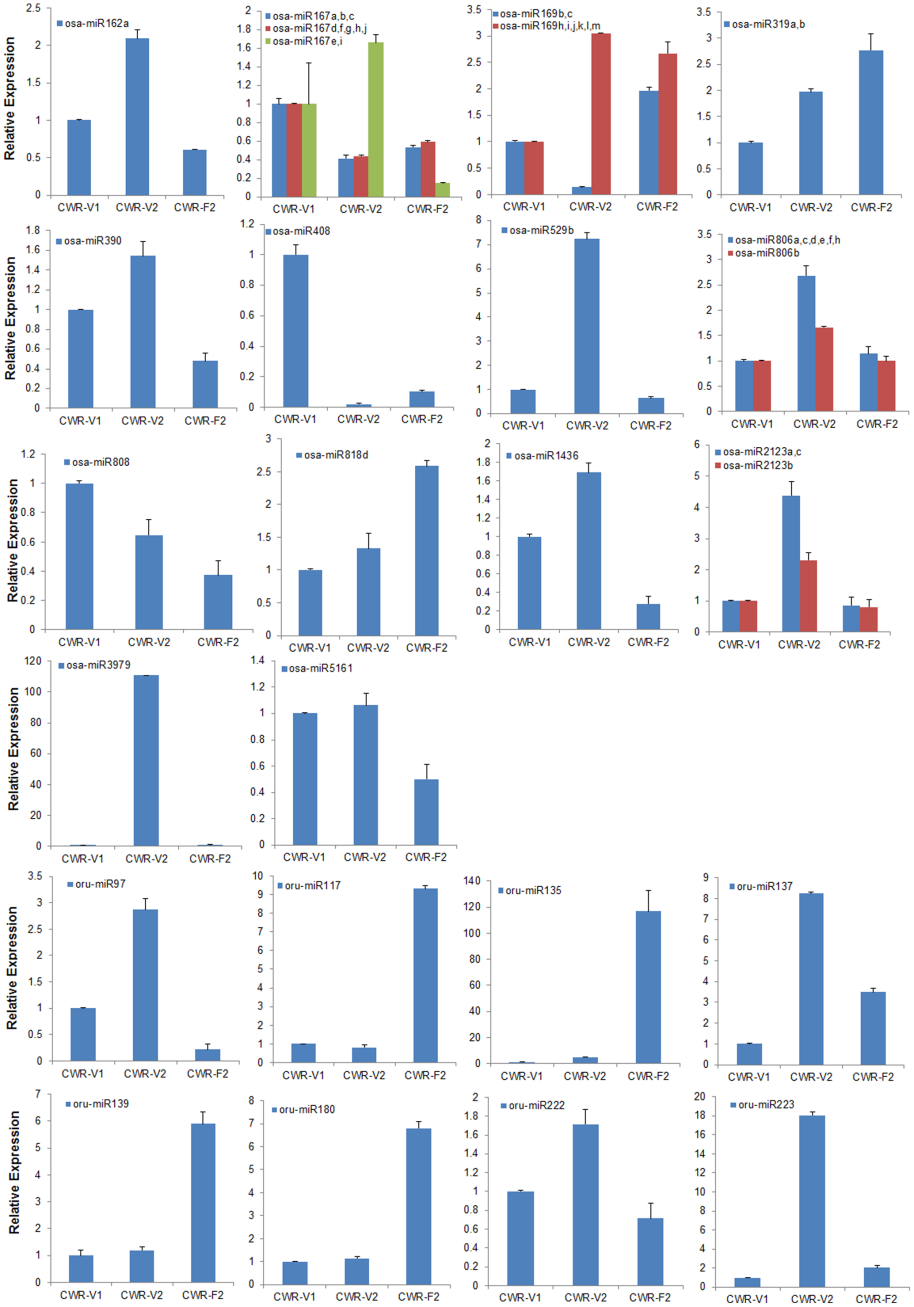
Quantitative real-time RT-PCR analysis of the relative expression of flowering miRNAs in the three sRNA libraries. The data represent the mean values ±SD of three replicates.

Based on the predicted function and expression of the miRNAs, we selected 47 out of the 95 flowering related miRNAs, corresponding to 22 families, for qRT-PCR, this included 14 known rice miRNAs families and eight new oru-miRNAs families (Figure 7). The flowering-related miRNAs abundance patterns in the three libraries as measured by qRT-PCR were compared with the deep sequencing data. Results showed that for 20 of the 22miRNA families, real-time PCR revealed the same expression patterns as did deep sequencing, despite some quantitative differences in expression levels. In consistent miRNA families include osa-miR818d and oru-miR222. The expression pattern of osa-miR818d in the deep sequencing is a quite bit difference with CWR-V1 = CWR-F2>CWR-V2 relationship, but in qPCR is CWR-F2>CWR-V2>CWR-V1.Similar to what was seen in the deep sequencing data, the expression of diverse miRNA members derived from a common family could be very different, but they had the same expression trends in the different libraries. For example, miR806, and miR2123 families. Unlike these two families, however, the miR167 and miR169 families showed different expression trends in the three libraries. MiR167a, b, c, d, f, g, h and j had the highest expression levels in CWR-V1, followed by CWR-F2 and CWR-V2, but the expression of miR167e and miR167i were highest in CWR-V2 and lowest in CWR-F2. In the miR169 family, the highest expression library for miR169b and miR169c was CWR-F2, this pattern differed from miR169h, i, j, k, l and m, which were expressed at their highest levels in CWR-V2. Single-library-expressed flowering miRNAs detected by deep sequencing that both expressed in all of the three libraries by qRT-PCR, and the read number of them was very low. In addition, the read numbers of miR169b,c, miR319, miR818d, oru-miR117, oru-miR135, oru-miR139, and oru-miR180 were highest in CWR-F2. The miRNAs with the lowest expression levels in CWR-F2 included osa-miR162a, miR167e,I, miR390, miR529b, miR808, miR1436, miR2123, miR5161, oru-miR97, oru-miR222, and oru-miR223. Most of these are F-miRNAs, their levels increased or declined during growth of the plants, and they controlled the expression of flowering targets (*SPL*, *TCP*, *MYB*, *AP2*, *WD*, etc.) and promoted flowering.

### Elucidation of flowering related miRNAs in *O. rufipogon*

At present, studies on the functional analysis of *O. rufipogon* genes primarily involve domestication, resistance to various stresses, and flowering variation. The rice domestication genes *SH4*, *qSH1*, *PROG1* and *OsSPL14* have been cloned [60], [61]. In general, plants of *O. rufipogon* flower once a year, the vegetative stage is as long as nine to ten months, and the flowering time last two to three months. Some variants flower twice a year, however. This different suggesting that the flowering mechanism must be different. Wang et al. [62] found 414 known rice miRNAs and 259 new specific miRNAs in *O. rufipogon* from Jiangxi Province, similar to this study, we identified some specific miRNAs in *O. rufipogon* that have become lost which, along with concomitant generation of new miRNAs, could contribute to rice evolution, in the meantime, a series of miRNAs related to flowering in *O. rufipogon* was identified and their differential expression patterns during the phase change from vegetative growth to flowering was analyzed.

Most of the 187 flowering related miRNAs are involved in the process of photoperiodic flowering, and a few affect floral organ formation(Figure S3). *SPL* transcriptional factors are regulated by osa-miR529b, oru-miR49, oru-miR139, oru-miR234, and oru-miR270, in addition to osa-miR156.The expression patterns for these miRNAs are similar, with the highest reads in CWR-V2, so they could have regulatory functions similar to miR156. Like miR172, osa-miR2096, osa-miR2125, oru-miR127, and oru-miR187 also target *AP2* genes. The target of oru-miR69, *Os01g08700*, is predicted to be a transcript encoding *OsGI*, which promotes photoperiodic flowering [63]. *Os07g30250*, the target of both miR1436 and miR818, is a homolog of *RFT1*, which has been reported to be a rice florigen, mediating rice flowering under long day conditions. Several rice genes have functions similar to *Arabidopsis CONSTANS* (*CO*) in regulating circadian rhythms; *Os01g10580.1*, *Os02g43170.1*, *Os02g43170.2*, *Os02g49880.1*, *Os04g45690.1*, *Os06g49880.2*, *Os06g49880.1*, and *Os05g11510.1*, are homologs of *Hd1* that activate *Hd3a* to promote flowering during short days and repress *Hd3a* to delay flowering during long days [64]. Their targets are negatively regulated by osa-miR818, osa-miR1848, osa-miR2123, oru-miR139, and oru-miR180. Osa-miR5161-targeted *Os05g47560.1* and osa-miR5512-targeted *Os07g31280.3* are the homologs of *Heading date 6* (*Hd6*), and can enhance *Hd1* repressor function in long days [65]. In addition to the genes discussed above, other circadian rhythm genes, including *ZTL*, *FKF*, *TOC1*, *PIF3* and *APR7/9*, are predicted to be targeted by flowering miRNAs in our study. Unlike the pleiotropy in photoperiodic flowering, miRNAs regulate the gibberellin (GA) flowering pathway mostly by repressing GA biosynthesis. For example, the targets of osa-miR531b, oru-miR33, oru-miR49, and oru-miR65 include *Os07g07420.1*, *Os01g66100.1*, *Os05g34854.1* and *Os05g34854.2*, which are predicted to encode gibberellin (GA)_20_-oxidase (GA_20_-ox). GA_20_-ox is a key enzyme that catalyzes the penultimate step in GA biosynthesis, the conversion of GA53 to GA20 [66]. Osa-miR531b and others miRNAs repress GA_20_-ox to block GA biosynthesis, regulating rice flowering. As discussed above, the complicated process of flowering in *O. rufipogon* is controlled by multiple genes, and metabolic mechanisms alone cannot regulate flowering without the function of miRNAs. Elucidation of the functions of these flowering related miRNAs not only depends on bioinformatic prediction, but also requires validation by conducting actual biological experiments.

## Conclusions

A total of three million unique sRNAs from >20 million clean reads were obtained from three deep sequencing datasets. 13.5% to 19.8% of the clean reads were mapped to known rice miRNAs, and 512 known rice miRNAs were identified. 290 new miRNAs that are specific to *O. rufipogon* was predicted, some of these were validated by cloning. In addition, functional annotation, GO, and KEGG pathway analysis revealed that 187 miRNAs were related to flowering or flowering variation in *O. rufipogon*. Based on the expression patterns of flowering related miRNAs in the vegetative and flowering stages, 95 flowering-related miRNAs was identified with significant differences. Among them, there are 66 flowering-related miRNAs, such as oru-miR97, oru-miR117,oru-miR135, oru-miR137, oru-miR139, osa-miR162a, osa-miR169b, osa-miR169c, oru-miR180, oru-miR222, oru-miR223, osa-miR319a-3p, osa-miR319b, osa-miR390, osa-miR408, osa-miR529b, osa-miR806a,b,c,d,e,f,h, osa-miR808, osa-miR818d, et al., and 17 early-flowering- related miRNAs, including osa-miR160f, osa-miR164d, osa-miR167d, f,g,h,I,j, osa-miR169a,k,l, osa-miR172b, osa-miR530-3p,5p, osa-miR818b,e, oru-miR4. Our study is the first to focus on flowering related miRNAs in *O. rufipogon*. We show that variation has occurred in miRNAs during rice evolution, and lay the foundation for further research into the molecular mechanisms of phase change and flowering in *O. rufipogon*.

## Supporting Information

Figure S1 **Number and distribution of sRNAs along the miRNA precursors.** (A) Examples of miRNA* from pre-miRNA were more abundant than those for the mature miRNA in CWR-F2 (the mature miRNA and its most abundant sRNA are shown in red and pink; the miRNA* and its most abundant sRNA are shown in blue and green, respectively). (B) Examples of multiple miRNAs generated from a single pre-miRNA in CWR-F2 (miRNAs are shown in red, pink and lilac, respectively).
(DOC)

Figure S2 Stem loop hairpin secondary precursor structures of 20 new miRNAs with complementary miRNA*. Segments that correspond to the mature miRNAs are shown in red, while the corresponding miRNA* sequences are shown in blue.
(TIF)

Figure S3 **The miRNA targets involved in plant circadian rhythms according to a KEGG pathway analysis.** The homologs of photoperiodic flowering genes as indicated by red boxes are shown with a blue bracket.
(TIF)

Table S1 **The primers used in this study.**
(XLSX)

Table S2 **Known rice miRNAs expressed in the common wild rice from our HTS datasets.**
(XLSX)

Table S3 **Newly identified miRNAs in the common wild rice from our HTS datasets.**
(XLSX)

Table S4 **Flowering targets prediction of flowering miRNAs from our HTS datasets.**
(XLSX)

Table S5 **Difference expression analyses of flowering miRNAs among the 3 libraries respectively.**
(XLSX)

Table S6 **The different expression of flowering miRNAs among the 3 libraries respectively**
(XLSX)
