# Supplementary material for: Identification and Functional Analysis of Flowering Related microRNAs in Common Wild Rice (Oryza rufipogon Griff.)
Source: PLoS One. 2013 Dec 30;8(12):e82844. doi: 10.1371/journal.pone.0082844 (PMC3875430; doi:10.1371/journal.pone.0082844)
Supplement: Figure S1 — Number and distribution of sRNAs along the miRNA precursors. (A) Examples of miRNA* from pre-miRNA were more abundant than those for the mature miRNA in CWR-F2 (the mature miRNA and its most abundant sRNA are shown in red and pink; the miRNA* and its most abundant sRNA are shown in blue and green, respectively). (B) Examples of multiple miRNAs generated from a single pre-miRNA in CWR-F2 (miRNAs are shown in red, pink and lilac, respectively). (DOC) [file pone.0082844.s001.doc]

Supplementary Figure S1. Number and distribution of sRNAs along the miRNA precursors.

**(A) Examples of miRNA* from pre-miRNA were more abundant than those for the mature miRNA in CWR-F2 (the mature miRNA and its most abundant sRNA are shown in red and pink; the miRNA* and its most abundant sRNA are shown in blue and green, respectively).**

**1、Pre-miR1425**

CUGUUGACUGCAUUAGGAUUCAAUCCUUGCUGCUAAAUGUAUUGCUUAUAUUCAGCAAUAUAAUGUUCAGCAGCAAGAACUGGAUCUUAAUAUAGUCGAUAG

((((((((((.(((((((((((...(((((((((((.(((((((((.......)))))))))...)).)))))))))...))))))))))).))))))))))

UUAGGAUUCAAUCCUUGCUGC_21_69 UUCAGCAGCAAGAACUGGAUC_21_4

UAGGAUUCAAUCCUUGCUGCU_21_888 UCAGCAGCAAGAACUGGAUCU_21_76

UAGGAUUCAAUCCUUGCUGCUA_22_30 UCAGCAGCAAGAACUGGAUCUUAA_24_1

UAGGAUUCAAUCCUUGCUGCUAA_23_23 CAGCAGCAAGAACUGGAUCUUAAU_24_9

UAGGAUUCAAUCCUUGCUGC_20_16 AGCAGCAAGAACUGGAUCUUA_21_5

UAGGAUUCAAUCCUUGCUG_19_13 AGCAGCAAGAACUGGAUCUUAAUA_24_4

UAGGAUUCAAUCCUUGCU_18_10 GCAGCAAGAACUGGAUCUUAA_21_12

UAGGAUUCAAUCCUUGCUGCUAAA_24_1 GCAGCAAGAACUGGAUCUUAAU_22_6

AGGAUUCAAUCCUUGCUGCU_20_6 CAGCAAGAACUGGAUCUUAAU_21_3021

AGGAUUCAAUCCUUGCUGCUA_21_2 CAGCAAGAACUGGAUCUUAAUA_22_34

AGGAUUCAAUCCUUGCUGCUAA_22_1 CAGCAAGAACUGGAUCUUAA_20_15

AUUCAAUCCUUGCUGCUAAAU_21_3 CAGCAAGAACUGGAUCUUA_19_2

AGCAAGAACUGGAUCUUAAUA_21_923

AGCAAGAACUGGAUCUUAAU_20_184

AGCAAGAACUGGAUCUUAA_19_9

AGCAAGAACUGGAUCUUA_18_4

AGCAAGAACUGGAUCUUAAUAU_22_1

GCAAGAACUGGAUCUUAAUA_20_1

AAGAACUGGAUCUUAAUA_18_1

**2、Pre-miR1846d**

UGCCUGUCCCUCCCACCGAGCAGCCGGAUCUCCCAGUGAGUAGGCCCGGGCCGCCAGAUCCGGUGACCCCGGUCUCCUCGCCGGUGUAUCCGGCGCCGCAGGGAGGGACGAGAGCC

.(((((((((((((..((.((.(((((((..(((.(((((.(((((.(((.((((......)))).))).))))).))))).)).).)))))))))))..))))))))).)).)).

UCCCACCGAGCAGCCGGAUCUC_22_8 UGUAUCCGGCGCCGCAGGGAGG_22_2

UCCCACCGAGCAGCCGGAUCUCC_23_1 GUAUCCGGCGCCGCAGGGAGG_21_24

UCCCACCGAGCAGCCGGAUCU_21_1 UAUCCGGCGCCGCAGGGAGG_20_7

AUCCGGCGCCGCAGGGAGG_19_2

**3、Pre-miR1853**

CGCAACUUCGAGCAUUCAAACAUUCCCAAUUACCAUCGCCUCCCUAAACAGCAAUGGUAAUUGGGGAUGUUCGGUUGCUCGAGGUUGCG

((((((((((((((..(.((((((((((((((((((.((...........)).)))))))))))))))))).)..))))))))))))))

UUCGAGCAUUCAAACAUUCCC_21_1 UAAUUGGGGAUGUUCGGUUGC_21_4

UCGAGCAUUCAAACAUUCCCA_21_1 UUGGGGAUGUUCGGUUGCU_19_1

**4、Pre-miR1860**

UCUUGAUUUGAUGGUAGGGUUAGUGUUAUUUGUAAAGAGAAAACCAGCUUCCAGAUCU-24nU-UCUUGUAGAUCUGGAAGCUAGGUUUUCUCUUUACAAAUAACACUAUUUUUUCCCCCAUUAGGUCUG

....((((((((((..(((.((((((((((((((((((((((((((((((((((((((-24nU-..))).))))))))))))).)))))))))))))))))))))))))......)))))))))))))..

UUGUAAAGAGAAAACCAGCU_20_2 UCUUGUAGAUCUGGAAGCUAGGUUU_25_3

UAAAGAGAAAACCAGCUUCC_20_1 UUGUAGAUCUGGAAGCUAGGUU_22_2

AAAGAGAAAACCAGCUUCCAG_21_1 AUCUGGAAGCUAGGUUUUCUCU_22_20

AGAAAACCAGCUUCCAGAUCU_21_2 AUCUGGAAGCUAGGUUUUCUC_21_1

UCUGGAAGCUAGGUUUUCUCUU_22_1

UCUGGAAGCUAGGUUUUCUCU_21_1

UGGAAGCUAGGUUUUCUCUUU_21_1

AAGCUAGGUUUUCUCUUUACAA_22_6

AAGCUAGGUUUUCUCUUUACA_21_3

AAGCUAGGUUUUCUCUUU_18_2

UUCUCUUUACAAAUAACACUA_21_1

**5、Pre-miR1866**

CUUUUGCACGGAGGGAUUUUGCGGGAAUUUCACGGGAAUUGAGUUGAUUCCUGAAAUUCCUGUAAAAUUCUUGUGUUCCAAAGGA

((((((.(((.(((((((((((((((((((((..((((((.....))))))))))))))))))))))))))).)))..)))))).

GAGGGAUUUUGCGGGAAUUUCACG_24_2 UGAAAUUCCUGUAAAAUUCUUGUG_24_10

GAGGGAUUUUGCGGGAAU_18_1 UGAAAUUCCUGUAAAAUUC_19_3

GAGGGAUUUUGCGGGAAUUU_20_1 UGAAAUUCCUGUAAAAUUCUUGU_23_3

GAGGGAUUUUGCGGGAAUUUCACGG_25_1

UGCGGGAAUUUCACGGGAAUUGAGU_25_1

**(B) Examples of multiple miRNAs generated from a single pre-miRNA in CWR-F2 (miRNAs are shown in red, pink and lilac, respectively).**

**1、Pre-miR159a**

10nt-UUGAGCUCCUUUCGGUCCAAAAAGGGGUGUUGCUGUGGGUCGAUUGAGCUGCUGGGUCAUGGAUCCC-81nt-AGGAAAAUGAUGGAGUACUCGU-12nt-CUUAUGGCUUGCAUGCCCCAGGAGCUGCAUCAACCCUACAUGGACCCUCUUUGGAUUGAAGGGAGCUCUGCAU

10nt-(.((((((((((((((((((..(((((.(((..(((((((.(((..((((.(((((.((((.(..((-81nt-.)))..))))))))..((....-12nt-)).))))..).)))).))))).))))..))).)))).)))..)))))))))))))))))))))))))).)).)

UUGAGCUCCUUUCGGUCCAAA_21_2 AGCUGCUGGGUCAUGGAUC_19_47 AGGAAAAUGAUGGAGUACUCGU_22_1 CUUGCAUGCCCCAGGAGCUGC_21_30 CCUCUUUGGAUUGAAGGGAGC_21_6

UUGAGCUCCUUUCGGUCCAAAA_22_1 AGCUGCUGGGUCAUGGAUCCC_21_8 UUGCAUGCCCCAGGAGCUGC_20_38 CUCUUUGGAUUGAAGGGAGC_20_1

UGAGCUCCUUUCGGUCCAAAA_21_7 AGCUGCUGGGUCAUGGAUCC_20_4 UUGCAUGCCCCAGGAGCUGCA_21_3 UCUUUGGAUUGAAGGGAG_18_141

UGAGCUCCUUUCGGUCCAAAAA_22_3 AGCUGCUGGGUCAUGGAU_18_2 UUGCAUGCCCCAGGAGCU_18_1 UCUUUGGAUUGAAGGGAGC_19_69

UGAGCUCCUUUCGGUCCAAA_20_1 GCAUGCCCCAGGAGCUGC_18_1 UCUUUGGAUUGAAGGGAGCU_20_8

GAGCUCCUUUCGGUCCAAAAA_21_10 CCAGGAGCUGCAUCAACCCUAC_22_1 UCUUUGGAUUGAAGGGAGCUC_21_1

GAGCUCCUUUCGGUCCAAA_19_2 CAGGAGCUGCAUCAACCCUACA_22_1 CUUUGGAUUGAAGGGAGC_18_514

GAGCUCCUUUCGGUCCAAAA_20_2 AGGAGCUGCAUCAACCCUACA_21_2 CUUUGGAUUGAAGGGAGCU_19_42

AGCUCCUUUCGGUCCAAAAAG_21_4 AUCAACCCUACAUGGACCCUC_21_10

AGCUCCUUUCGGUCCAAA_18_1 AUCAACCCUACAUGGACCCUCU_22_4

AGCUCCUUUCGGUCCAAAAA_20_1 AUCAACCCUACAUGGACCCU_20_2

AGCUCCUUUCGGUCCAAAA_19_1 CUUUGGAUUGAAGGGAGCUCU_21_3

GGGGUGUUGCUGUGGGUCGAUUGA_24_1 UUUGGAUUGAAGGGAGCU_18_25680

GGGUGUUGCUGUGGGUCGAUUG_22_1 UUUGGAUUGAAGGGAGCUCUG_21_570

UUUGGAUUGAAGGGAGCUC_19_329

UUUGGAUUGAAGGGAGCUCU_20_63

UUUGGAUUGAAGGGAGCUCUGC_22_20

UUUGGAUUGAAGGGAGCUCUGCA_23_3

UUGGAUUGAAGGGAGCUCUGC_21_20

UUGGAUUGAAGGGAGCUCUG_20_4

UUGGAUUGAAGGGAGCUC_18_3

UUGGAUUGAAGGGAGCUCUGCA_22_2

UUGGAUUGAAGGGAGCUCU_19_1

UGGAUUGAAGGGAGCUCUGC_20_3

UGGAUUGAAGGGAGCUCUGCA_21_1

UGGAUUGAAGGGAGCUCUG_19_1

UGGAUUGAAGGGAGCUCU_18_1

**2、Pre-miR169f**

GGGCCUUCCAUGAGGACAAGAGCUGAUUCGGUAGCCAAGGAUGACUUGCCUAAUGCCUA-102nt-AGGCAUGUCUUCCUUGGCUAUUCGGAGCGGCUCUUGUCUCUCGUGGAAGGCUG

.((((((((((((((((((((((((.(((((((((((((((.(((.((((.(((((..(-102nt-))))).))).)))))))))).))))).))))))))))).))))))))))))).

UGAGGACAAGAGCUGAUUCGG_21_1895 AGGCAUGUCUUCCUUGGCUAU_21_1

UGAGGACAAGAGCUGAUUCGGU_22_18 GUCUUCCUUGGCUAUUCGGAGCGG_24_1

UGAGGACAAGAGCUGAUUC_19_1 CUUGGCUAUUCGGAGCGGCUCU_22_1

GAGGACAAGAGCUGAUUCGG_20_3 UUGGCUAUUCGGAGCGGCUCU_21_1

AGGACAAGAGCUGAUUCGGUA_21_17 UUGGCUAUUCGGAGCGGCUCUUGUC_25_1

AGGACAAGAGCUGAUUCGGUAGCC_24_8

AGGACAAGAGCUGAUUCGGUAGC_23_5

AGGACAAGAGCUGAUUCGGUAGCCA_25_1

AGGACAAGAGCUGAUUCGG_19_1

AGCUGAUUCGGUAGCCAAGGAUG_23_1

AGCUGAUUCGGUAGCCAAGGA_21_1

UAGCCAAGGAUGACUUGCCU_20_3

UAGCCAAGGAUGACUUGCCUA_21_1

UAGCCAAGGAUGACUUGC_18_1

AGCCAAGGAUGACUUGCC_18_1

**3、Pre-miR1863b**

CAAUAGCAAUGCAUCAGUUACGUUUCCUACAUAGUUACAUGGUAUCAGAGCUG-233nt-UGAGGAUCUUAGCUGUUAGCUCUGAUACCAUGUUAACUGUUUAGAGACUUGGCUGAUGCAUUACUUUUGGAAAC

(((.((.(((((((((((((.(((((.((.(((((((((((((((((((((((-233nt-).)))))........))))))))))))))))).))))))).))))))).))))))))))))).)).))).....

AUAGUUACAUGGUAUCAGAGC_21_1 AGCUCUGAUACCAUGUUAACUGUU_24_1

UAGAGACUUGGCUGAUGCAUUACU_24_26

UAGAGACUUGGCUGAUGCAUUAC_23_6

UAGAGACUUGGCUGAUGCAUUACUU_25_1

AGAGACUUGGCUGAUGCAUUACUU_24_59

AGAGACUUGGCUGAUGCAUUACU_23_48

AGAGACUUGGCUGAUGCAUUAC_22_7

**4、Pre-miR2864**

UCUAUUCAGCUUUUGCUGCCCUUGUUUUGCAUUGUAUAGGUAUAGCAAUAUGCCAUUCUGUAGUGGCAUACCUCUACAGUGCAAAACAAGGACAACAAGAG

.........((((((.((.(((((((((((((((((.(((((........((((((......))))))))))).))))))))))))))))).)).))))))

UUUUGCUGCCCUUGUUUUGCA_21_1 ACAGUGCAAAACAAGGACAACAAG_24_2

UUGCUGCCCUUGUUUUGCAUU_21_6 AGUGCAAAACAAGGACAACAA_21_6

UGCUGCCCUUGUUUUGCAUU_20_1 AAAACAAGGACAACAAGA_18_1

UUGUUUUGCAUUGUAUAGGUA_21_42

UUUUGCAUUGUAUAGGUAUAG_21_2

**5、Pre-miR319a**

-27nt-CACUCUCAGAUGGCUGUAGGGUUUUAUUAGCUGCCGAAUCAUCCAUUCA-40nt-ACUGGAUGACGCGGGAGCUAAAAUUUAGCUCUGCGCCGUUUGUGGUUGGACUGAAGGGUGCUCCCUUGCUCAAGC

-27nt-((..(.((((((((.((((((((...((((((.(((..(((((((.((.-40nt-).)))))))..))).)))))).....)))))))))))))))).)..)))))))))))).)))))))))).))...

UAGCUGCCGAAUCAUCCAUUC_21_1 ACUGGAUGACGCGGGAGCUAA_21_88 UUGGACUGAAGGGUGCUCCCU_21_77

ACUGGAUGACGCGGGAGCUAAA_22_14 UUGGACUGAAGGGUGCUCC_19_3

ACUGGAUGACGCGGGAGCUAAAA_23_1 UUGGACUGAAGGGUGCUCCC_20_3

ACUGGAUGACGCGGGAGCU_19_1 UUGGACUGAAGGGUGCUC_18_2

CUGGAUGACGCGGGAGCUAA_20_1 GGACUGAAGGGUGCUCCCU_19_1

**6、Pre-miR444a**

AUGCAAUUGGGGGCAGCAAGCUAGAGGUGGCAACUGCAUAAUUUGCAAGAAAUUGUUGGCUGAAGAUCAUACCGAUGAUAUUCUUGCAAGUUAUGCAGUUGCUGCCUCAAGCUUGCUGCCUCCUGUUGCCA

..(((((.((((((((((((((.((((..((((((((((((((((((((((((..((((.((.....))..))))..)).))))))))))))))))))))))..)))).)))))))))))))).)))))..

UUGGGGGCAGCAAGCUAGAGGUGG_24_1 AUUUGCAAGAAAUUGUUGGCUGAAG_25_1 CUUGCAAGUUAUGCAGUUGCUGCC_24_3

AAGCUAGAGGUGGCAACUGCA_21_1 CUUGCAAGUUAUGCAGUUGCUGC_23_2

AGCUAGAGGUGGCAACUGCAU_21_417 GCAAGUUAUGCAGUUGCUGCCUCA_24_3

AGCUAGAGGUGGCAACUGCA_20_118 CAAGUUAUGCAGUUGCUGCCU_21_27

AGCUAGAGGUGGCAACUGCAUA_22_29 AAGUUAUGCAGUUGCUGCCUC_21_2

AGCUAGAGGUGGCAACUGCAUAA_23_2 AAGUUAUGCAGUUGCUGCCUCA_22_1

AGCUAGAGGUGGCAACUGCAUAAU_24_1 AGUUAUGCAGUUGCUGCCUCA_21_10

GCUAGAGGUGGCAACUGCAUA_21_585 AGUUAUGCAGUUGCUGCCUCAAGC_24_1

GCUAGAGGUGGCAACUGCAU_20_42 UUAUGCAGUUGCUGCCUCAAGC_22_1

GCUAGAGGUGGCAACUGCAUAAUU_24_4 UAUGCAGUUGCUGCCUCAAGCUUG_24_1

GCUAGAGGUGGCAACUGCAUAA_22_1 AUGCAGUUGCUGCCUCAAGCU_21_5

GCUAGAGGUGGCAACUGCA_19_1 AUGCAGUUGCUGCCUCAAGC_20_4

CUAGAGGUGGCAACUGCAUAA_21_8 AUGCAGUUGCUGCCUCAAGCUU_22_2

CUAGAGGUGGCAACUGCAUA_20_2 UGCAGUUGCUGCCUCAAGCUU_21_1123

UAGAGGUGGCAACUGCAUAAUU_22_48 UGCAGUUGCUGCCUCAAGCU_20_4

UAGAGGUGGCAACUGCAUAAU_21_28 UGCAGUUGCUGCCUCAAGC_19_2

UAGAGGUGGCAACUGCAUAA_20_13 UGCAGUUGCUGCCUCAAG_18_2

UAGAGGUGGCAACUGCAUA_19_1 GCAGUUGCUGCCUCAAGCUUG_21_12

UAGAGGUGGCAACUGCAUAAUUUG_24_1 GCAGUUGCUGCCUCAAGCUU_20_2

AGAGGUGGCAACUGCAUAAUU_21_13 UUGCUGCCUCAAGCUUGCUGC_21_5

AGAGGUGGCAACUGCAUAAU_20_1 UUGCUGCCUCAAGCUUGCUGCC_22_1

AGGUGGCAACUGCAUAAUUUGCAA_24_5 UGCUGCCUCAAGCUUGCUGCC_21_10

AGGUGGCAACUGCAUAAUUUG_21_4

GGUGGCAACUGCAUAAUUUGCAAG_24_1

UGGCAACUGCAUAAUUUGCAAGAA_24_2

**7、Pre-miR444d**

AGUUAUUGCACAUGGUGGCACCAAGCAUGAGGCAACAACUGCAUUACUUGCAAGAAAGGCACAAAAUCAUUAGAUGAUUACUUGUGGCUUUCUUGCAAGUUGUGCAGUUGCUGCCUCAAGCUUGCUGCCUCCCUCUGCCAAAU

.......(((...((.((((.(((((.(((((((.(((((((((.(((((((((((((.(((((((((((...))))))..))))).))))))))))))).))))))))).))))))).))))).)))).))...))).....

AUUACUUGCAAGAAAGGCACA_21_4

UUACUUGCAAGAAAGGCACAA_21_1

UACUUGCAAGAAAGGCACAAA_21_2

ACUUGCAAGAAAGGCACAAAA_21_8

UUGUGGCUUUCUUGCAAGUUG_21_22

UUUCUUGCAAGUUGUGCAGUU_21_10

UUCUUGCAAGUUGUGCAGUUG_21_4

UUCUUGCAAGUUGUGCAGUU_20_1

UGCAAGUUGUGCAGUUGCUGC_21_1

UGCAGUUGCUGCCUCAAGCUU_21_1123

UGCAGUUGCUGCCUCAAGCU_20_4

UGCAGUUGCUGCCUCAAGC_19_2

UGCAGUUGCUGCCUCAAG_18_2

GCAGUUGCUGCCUCAAGCUUG_21_12

GCAGUUGCUGCCUCAAGCUU_20_2

UUGCUGCCUCAAGCUUGCUGC_21_5

UUGCUGCCUCAAGCUUGCUGCC_22_1

UGCUGCCUCAAGCUUGCUGCC_21_10
