# Supplementary figures and images for: Identification and Functional Analysis of Flowering Related microRNAs in Common Wild Rice (Oryza rufipogon Griff.)
Source: PLoS One. 2013 Dec 30;8(12):e82844. doi: 10.1371/journal.pone.0082844 (PMC3875430; doi:10.1371/journal.pone.0082844)

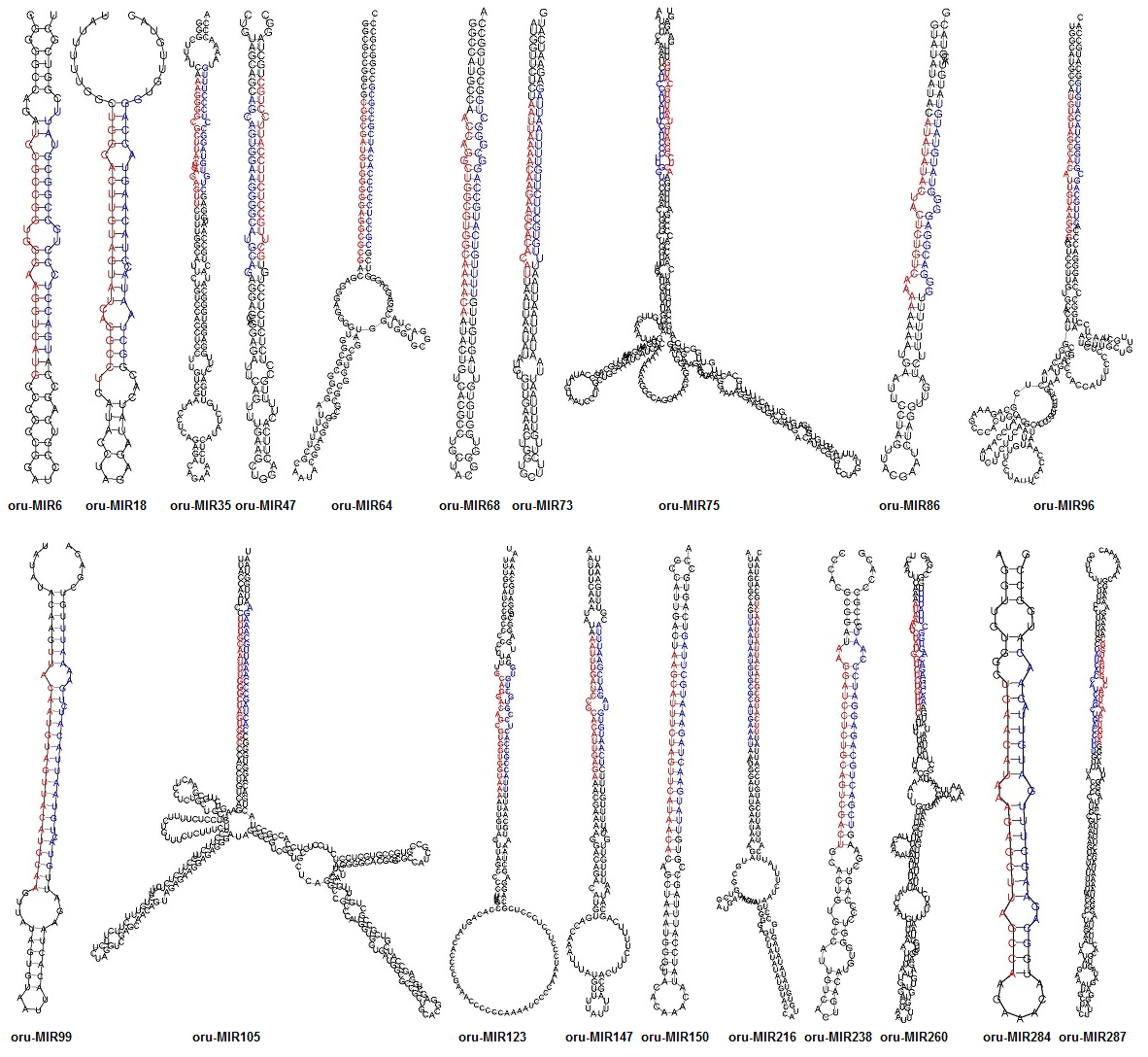

Supplement: Figure S2 — Stem loop hairpin secondary precursor structures of 20 new miRNAs with complementary miRNA*. Segments that correspond to the mature miRNAs are shown in red, while the corresponding miRNA* sequences are shown in blue. (TIF) [file pone.0082844.s002.tif]

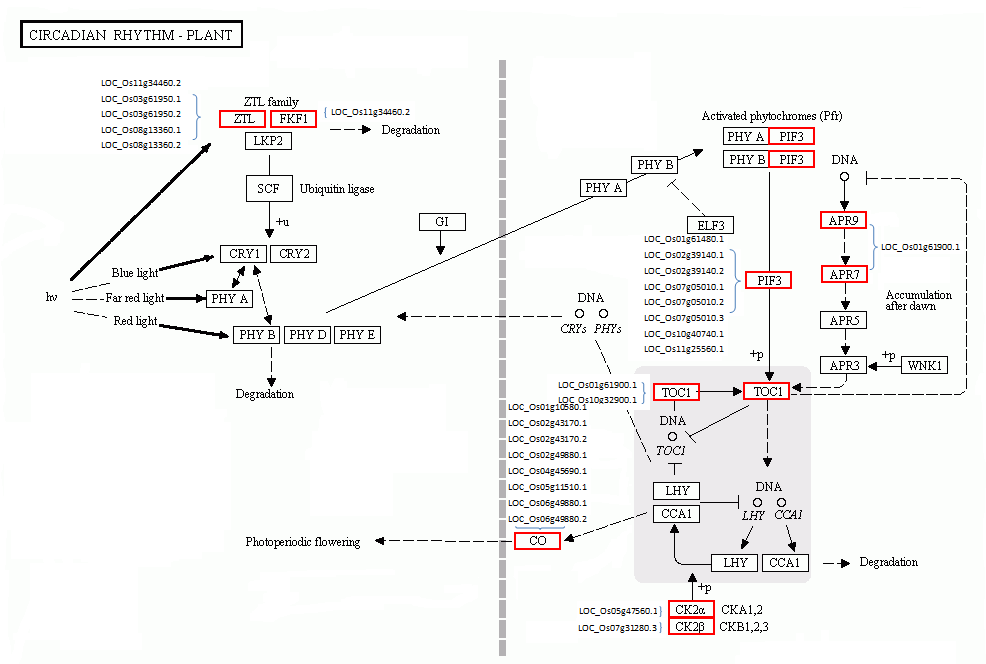

Supplement: Figure S3 — The miRNA targets involved in plant circadian rhythms according to a KEGG pathway analysis. The homologs of photoperiodic flowering genes as indicated by red boxes are shown with a blue bracket. (TIF) [file pone.0082844.s003.tif]
